# Localized Query Attack Toward Transformer-Based Visible Object Detectors

**DOI:** 10.3390/s26061987

**Published:** 2026-03-23

**Authors:** Yang Wang, Ang Li, Zhen Yang, Xunyun Liu

**Affiliations:** Academy of Military Sciences, Beijing 102205, China

**Keywords:** adversarial patch, computer vision, object detection, transformer models, attention mechanisms

## Abstract

Transformer-based detectors have demonstrated exceptional accuracy in visible-object detection tasks. However, adversarial patches, specific types of adversarial examples, can disrupt these detectors by introducing unrestricted perturbations into specific image regions. Traditional methodologies focus on placing patches directly on objects and increasing attention scores between the patch and all areas of the image to impair detector performance. Nevertheless, these approaches are suboptimal due to significant discrepancies between background and object features, which contradict optimization objectives. Moreover, they overlook the impact of cross-attention mechanisms on detection results. To address these limitations, we introduce a novel approach named Localized Query Attack (LQA), designed to interfere with both self-attention within the encoder and cross-attention in the decoder. Unlike conventional global interference methods, LQA targets object features specifically, enhancing self-attention interactions between the adversarial patch and foreground regions to redirect model focus toward the patch. In the context of decoder cross-attention, we compute the joint attention matrix connecting encoder outputs with object queries. By diminishing the influence of encoder outputs and residual components in this matrix, we amplify the relative importance of the adversarial patch, thereby intensifying the attack’s effectiveness. Our experiments show that LQA achieves an approximately 20% improvement in transfer attack performance compared to the second-best method across various transformer-based detectors. The practical efficacy of LQA is further substantiated through real-world scenario validations, underscoring its applicability.

## 1. Introduction

The transformer architecture was originally developed for natural language processing tasks. Subsequently, it has been successfully adapted to computer vision tasks, achieving performance comparable to that of Convolutional Neural Network (CNN)-based detectors in areas such as image classification [1,2,3] and object detection [4,5,6,7,8,9,10].

Transformer-based detectors primarily rely on self-attention and cross-attention mechanisms. These detectors employ a backbone network to extract image features, which are subsequently processed by the encoder’s self-attention mechanism in the form of a token sequence. In the self-attention mechanism, the three inputs—query (Q), key (K), and value (V)—are all derived from the image features extracted by the backbone network. The decoder consists of both self-attention and cross-attention mechanisms, which share a similar structural design but differ in their input sources. Within the decoder, the Q, K, and V for the self-attention mechanism are learnable parameters commonly referred to as “object queries.” Each object query represents a potential object to be detected.

**Figure 1:**
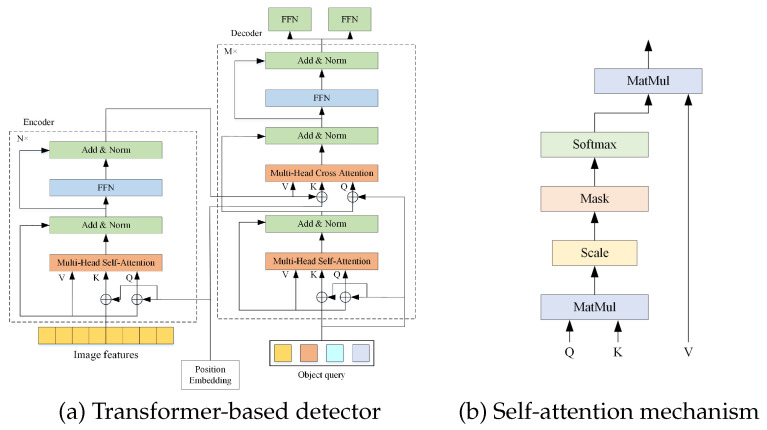
Overview of the transformer-based detection framework. (**a**) shows the end-to-end architecture; (**b**) illustrates the self-attention computation in the encoder.

The token sequence generated by the self-attention mechanism in the decoder, along with the output tokens from the encoder, is used to compute cross-attention, which ultimately contributes to the generation of detection results. Within the detector, self-attention primarily captures contextual relationships among tokens in the sequence, enabling a global understanding of object information. Cross-attention, on the other hand, aligns object queries with the encoder’s aggregated feature representations. The overall architecture of a typical transformer-based detector is illustrated in Figure 1.

Although transformer-based detectors and CNN-based detectors differ significantly in architectural design, both are susceptible to adversarial patches [11,12]. First introduced by Brown et al. [13], adversarial patches refer to localized, unrestricted perturbations added to input images, which can cause Deep Neural Network (DNN) models to produce incorrect outputs. A substantial body of research has demonstrated that adversarial patches generated using a white-box surrogate model can effectively mislead black-box models with unknown parameters and architectures—a property known as attack transferability [14,15,16]. Assessing transformer-based detectors through the lens of adversarial patch transferability provides valuable insights into their security vulnerabilities, supports the development of more robust models for real-world deployment, and has garnered increasing attention within the academic community.

The integration of transformer-based object detectors into modern vision sensor systems—ranging from autonomous vehicles and surveillance cameras to mobile robotics and wearable devices—has accelerated their deployment in safety-critical applications. However, as these models become embedded in edge-AI sensor pipelines, their susceptibility to adversarial perturbations introduces tangible risks to system reliability and operational safety. Recent studies have emphasized the need for robustness-aware design in vision-based sensing, particularly under physical-world conditions where lighting variations, occlusions, and sensor noise are unavoidable [17,18]. Our work directly addresses this emerging concern by exposing critical vulnerabilities in attention mechanisms that are now foundational to many on-device detection frameworks. By demonstrating that localized adversarial patches can reliably degrade detector performance across diverse transformer architectures—even under real-world sensing constraints—this paper contributes to the growing field of secure and trustworthy sensing.

A number of studies have aimed to disrupt the self-attention mechanism within the encoder, seeking to manipulate the attention weights such that malicious query tokens are prioritized, thereby inducing erroneous detector outputs [19,20]. However, we observe that such approaches may not be fully effective, as adversarial perturbations often fail to align with the semantic structure of the input. In the multi-head self-attention layer of the encoder, the attention weight $a(x_{i},\;x_{j})$ between the *i*-th and *j*-th tokens is computed as

(1) $$a\left(x_{i},x_{j}\right)=\frac{x_{i}W_{Q}{\left(x_{j}Wk\right)}^{T}}{{\sqrt{d}}_{k}}=\frac{q_{i}k_{j}^{T}}{{\sqrt{d}}_{k}}$$

Let the input token sequence be denoted as x = $\left[x_{0},\;x_{1},\;\ldots ,\;x_{J}\right]$, where each $x_{i}\in R^{d}$ represents a token embedding. The learnable projection matrices for query (Q), key (K), and value (V) are denoted as $W^{Q}$, $W^{K}$, and $W^{V}$, respectively. The attention score between the *i*-th and *j*-th tokens is abbreviated as $a_{ij}$, which is obtained by applying the softmax function to the raw attention logits:

(2) $$a_{ij}=\mathrm{softmax}\left(\frac{\left(x_{i}W^{Q}\right){\left(x_{j}W^{K}\right)}^{⊤}}{\sqrt{d_{k}}}\right).$$

The output of the self-attention mechanism at the *i*-th position is computed based on a weighted aggregation of all input tokens, formulated as

(3) $$z_{i}=\sum_{j=0}^{J}a_{ij}\left(x_{j}W^{V}\right)=\sum_{j=0}^{J}a_{ij}v_{j},$$

where $v_{j}=x_{j}W^{V}$ denotes the value vector associated with the *j*-th token.

From Equation (3), it is evident that the output of self-attention at position *i* is a weighted sum of features from all positions in the input sequence. In the context of images, significant discrepancies exist between foreground (i.e., object) and background features, as well as among features corresponding to different object classes. This heterogeneity poses a challenge in training a single adversarial patch that can effectively interfere with both object and background tokens, which may exhibit vastly different feature distributions.

**Figure 2:**
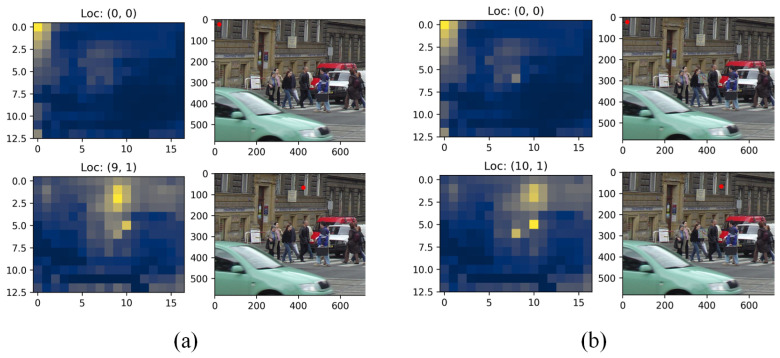
Visualization of (**a**) original self-attention map and (**b**) adversarial self-attention map.

As shown in Figure 2a, the visualization of the self-attention map within the red region indicates that attention scores decrease with increasing spatial distance from the query location, while higher scores are observed among tokens belonging to the same object in adjacent regions. In Figure 2b, we apply an adversarially trained patch designed to amplify attention scores between the patch and other positions. The visualization of the red area reveals a more pronounced change in attention scores near the adversarial patch, suggesting stronger interference in its vicinity.

These results indicate that the patch exerts greater influence on nearby regions. However, increasing the attention weights between the adversarial patch and both object and background features across the image remains a challenging task. Furthermore, the original encoder computes self-attention over the entire input sequence, resulting in high computational complexity. To address this issue, subsequent works have focused on simplifying the encoder architecture through techniques such as sparse self-attention and lightweight network designs. These approaches often generate only a limited number of query tokens, primarily concentrated around object regions.

The architectural variations among different transformer-based detectors—particularly in terms of encoder design—lead to inconsistent attention behaviors, which in turn hinder the transferability of adversarial patches across models.

Moreover, these methods do not affect the cross-attention mechanism within the decoder. The output of cross-attention is formed by combining the encoder’s output tokens with a residual component derived from the object query. Enhancing the influence of adversarial tokens propagated from the encoder through the cross-attention mechanism may offer a promising avenue for improving attack effectiveness.

**Figure 3:**
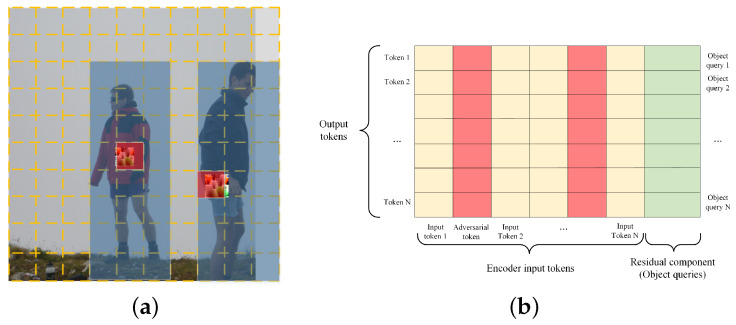
(**a**) Attack on encoder mechanism. (**b**) Joint attention matrix (JAM).

In this paper, we propose the Localized Query Attack (LQA), a targeted adversarial attack specifically designed for transformer-based object detectors. LQA utilizes an adversarial patch with unrestricted perturbations to disrupt both the encoder and decoder components of the model. Within the encoder, LQA interferes with the self-attention mechanism by selectively amplifying attention scores between object regions and the adversarial patch. As illustrated in Figure 3a, the red area highlights the location of the adversarial token in the input image. LQA focuses on strengthening the self-attention interactions between the adversarial token and the surrounding object region (indicated in blue). Due to the high feature similarity in the vicinity of the object, LQA aligns more coherently with the inherent attention mechanism compared to global interference strategies. This alignment leads to more effective disruption of the encoder’s representation learning, as reflected in subsequent performance degradation.

Inspired by the work of Ferrando et al. on transformer interpretability in text translation tasks [21], we compute the joint attention matrix (JAM) for the decoder. This matrix decomposes the cross-attention output into contributions from distinct source tokens, as illustrated in Figure 3b. By attenuating the contributions of residual and normal encoder tokens within the JAM, we amplify the influence of the adversarial token, thereby effectively disrupting the cross-attention mechanism.

To evaluate the efficacy of LQA, we conducted experiments against five state-of-the-art methods using five different transformer-based detectors across two datasets. Our results demonstrate a significant improvement, achieving up to an 18.38% enhancement over the second-best method. Additionally, we validate the practical applicability of LQA in real-world scenarios, aiming to bolster the security applications of transformer-based detectors. Our research not only contributes theoretically but also showcases the potential of LQA in enhancing model robustness and security.

## 2. Background

This work involves two aspects: the transformer-based object detector and adversarial patch.

### 2.1. Transformer-Based Object Detector

Object detection is a fundamental task in computer vision, aiming to simultaneously localize and classify objects within an image. Deep Neural Networks (DNNs) have significantly accelerated progress in this domain. Early DNN-based object detection models were primarily built upon CNN [10], achieving high accuracy and inference speed. However, these methods often relied on manually designed components, such as region proposal networks or anchor mechanisms, leading to complex architectures that hindered fully end-to-end training [22,23,24].

To address these limitations, transformer-based detectors have been introduced. Among them, the DEtection TRansformer (DETR) [4] serves as a representative framework. DETR formulates object detection as a set prediction problem, employing an encoder–decoder architecture. The encoder captures global contextual information through self-attention mechanisms, while the decoder performs cross-attention between encoder features and learnable object queries to generate detection results. The simplicity of DETR’s design enables end-to-end learning, marking a milestone in the application of transformers to object detection.

Despite its architectural elegance, DETR suffers from issues such as slow convergence and computational inefficiency. To mitigate these challenges, recent works have proposed structural improvements. Zhu et al. [18] modified the encoder by generating sparse query tokens over multi-scale feature maps, thereby improving both convergence speed and detection accuracy. Wang et al. [25] pointed out that the object queries in the original decoder lack explicit spatial interpretation, which hampers optimization as they do not inherently focus on specific image regions. To address this, they introduced anchor points into the decoder, guiding object queries toward relevant spatial locations and accelerating convergence.

Meng et al. [26] addressed the same issue by decoupling object queries into content and spatial components. This approach aims to improve convergence by reducing dependency on content queries while enhancing the effectiveness of spatial queries. DAB-DETR [27] further builds on this idea by using anchor boxes as object queries and updating them dynamically across layers. This method not only accelerates convergence but also adapts positional feature maps according to box dimensions, thereby improving overall detection performance. Additionally, Li et al. [28] tackled the instability caused by random initialization in bipartite matching during DETR’s training phase. They proposed Query DeNoising, a novel training strategy in which noisy anchor boxes are used as decoder queries. Beyond detection, these queries participate in a denoising process to stabilize training and enhance model precision.

These advancements have progressively alleviated DETR’s initial shortcomings in convergence speed and small-object detection accuracy. However, they also involve substantial modifications to the original DETR architecture—particularly in the design of the encoder and decoder modules. These architectural variations among different transformer-based detectors lead to diverse attention behaviors, posing significant challenges for the transferability of adversarial attacks across models.

### 2.2. Adversarial Attack

Szegedy et al. [29] identified an intriguing property of Deep Neural Networks (DNNs): the introduction of imperceptible perturbations to input data can cause DNN-based models to produce incorrect outputs. These manipulated inputs are referred to as adversarial examples. Subsequent studies have revealed that adversarial examples generated using a white-box model as a surrogate can still effectively mislead black-box models with unknown architectures and parameters [16]. This phenomenon, known as attack transferability, highlights significant security vulnerabilities in DNN-based systems.

Brown et al. [13] first introduced the concept of adversarial patches. Unlike earlier adversarial examples that apply small, global perturbations across the entire image, adversarial patches modify specific localized regions without constraining the magnitude of pixel changes. This approach opened new directions in adversarial attack research.

Most prior works on adversarial patch attacks have focused on CNN-based object detectors. Liu et al. [30] proposed DPATCH, which inserts adversarial patches into specific image regions to evade detection. Lee et al. [31] extended adversarial patches to the physical world by placing them in real scenes to cause object detectors to miss objects entirely. Huang et al. [32] improved transferability through model self-ensembling using Shakedrop. Hu et al. [33] employed a pretrained generative model to synthesize visually realistic adversarial patches.

Recent efforts have explored adversarial patch attacks against transformer-based models. Wei et al. [34] enhanced transferability by suppressing gradient magnitudes through attention matrix manipulation. Zhang et al. [35] proposed Token Gradient Regularization to stabilize optimization by eliminating extreme gradients. In Patch-Fool, Fu et al. [19] introduced an attention-aware loss function that disrupts detection by increasing self-attention scores between the adversarial patch and other regions, while mitigating gradient conflicts via PCGrad. Attention-Fool [36] trained adversarial patches by minimizing the distance between key and query features. Zhu et al. [37] scaled mild gradients to reduce overfitting to surrogate models.

Despite these advances, existing adversarial patch methods for transformer-based models remain limited and primarily focus on perturbing the self-attention mechanism. They largely overlook the architectural diversity among different detector designs, which significantly hampers cross-model attack transferability. Moreover, few studies have investigated the impact of cross-attention mechanisms in DETR-based architectures—despite their critical role in object query generation and feature fusion.

## 3. Localized Query Attack

### 3.1. Preliminaries

LQA trains adversarial patches using DETR as a local surrogate white-box detector *f*. Given an input dataset x∈X, the objective of LQA is to optimize the adversarial patch δ such that the following condition is satisfied:

(4) f(x+δ)≠y,

where $\delta \in {\left[0,\;1\right]}^{H}\times^{W}\times^{C}$ denotes the adversarial patch applied to the input image, and *y* represents the detection output of the model. The detection result for the *i*-th object is defined as $y^{\left(i\right)}\;=\;\left\{B^{\left(i\right)},\;S^{\left(i\right)}\right\}$, where $B^{\left(i\right)}\;=\;\left\{x,\;y,\;w,\;h\right\}$ corresponds to the bounding box with top-left coordinates (x, y) and dimensions (w, h), and $S^{\left(i\right)}$ denotes the classification score (before softmax) associated with the detected object. We denote the ground truth annotations as *G*.

### 3.2. Method

LQA is primarily composed of two loss components: $L_{\mathrm{De}}$, which targets the self-attention mechanism in the encoder, and $L_{\mathrm{En}}$, which focuses on the cross-attention mechanism within the decoder.

#### 3.2.1. Attack on Encoder

Several prior works attempt to disrupt the self-attention mechanism by either increasing the discrepancy in attention weights between adversarial examples and clean images, or amplifying the self-attention scores between adversarial tokens and other tokens. However, we identify two major limitations in these approaches. First, the significant feature variations among different tokens make it difficult to optimize a single adversarial patch that can effectively disrupt both object and background tokens simultaneously. Second, substantial architectural differences exist among the encoders of various transformer-based detectors. As a result, perturbing all tokens indiscriminately does not align well with the design trends of more advanced models.

To address these issues, we propose LQA to locally disrupt the self-attention within the encoder, as illustrated in Figure 3a. In the figure, the yellow grid represents distinct image regions corresponding to individual tokens mapped back to the input space, while the red region highlights the location of adversarial tokens introduced by the adversarial patch. The blue box indicates the detected object region. We denote the set of adversarial tokens as $x^{\mathrm{adv}}$, and the set of object tokens within the detection box as $x^{\mathrm{obj}}$. Based on this formulation, the loss term for disrupting self-attention in the encoder is defined as $L_{\mathrm{En}}$.

(5) $$L_{En}=-\sum_{j=0}^{J}\sum_{i=0}^{I}a\left(x_{j}^{adv},x_{i}^{obj}\right)$$

We aim to disrupt the detector by increasing the self-attention scores between adversarial tokens and normal tokens within the corresponding detection boxes. Tokens located inside the same detection box typically share similar visual features, as they belong to a single object instance. When an adversarial patch occupies any part of the detection box, the resulting adversarial token should exhibit heightened self-attention interactions with other tokens in that region. Therefore, we randomly select the position of the adversarial patch within the detection box during training. The patch placement is determined according to the following formulation:

(6) $$\mathrm{Location}=\left(x+\lambda_{x}\cdot w,\;y+\lambda_{y}\cdot h\right),\;\lambda_{x},\lambda_{y}∼U\left(0,1\right)$$

Let (x,y) denote the coordinates of the top-left corner of the detection box, and let (w,h) represent its width and height, respectively. The coefficients $\lambda_{x}$ and $\lambda_{y}$ are independently sampled from a uniform distribution over the interval (0, 1), ensuring diverse and spatially balanced placement of the adversarial patch within the detection box.

#### 3.2.2. Attack on Decoder

The decoder layer in transformer-based architectures primarily consists of self-attention and cross-attention mechanisms, which share a similar structural framework but differ in their input sources. In the self-attention mechanism, the query, key, and value vectors are all derived from the decoder’s learnable object queries. Initially, self-attention is computed among these object queries to model inter-object dependencies and contextual relationships. Subsequently, the image features extracted by the encoder serve as the key and value inputs, while the output of the self-attention module acts as the query for the cross-attention mechanism. This enables the decoder to selectively extract object-related information from the global image features. The final output tokens are generated by integrating the output of the cross-attention module with the original query through residual connections, where this fused representation serves as the input for subsequent decoder layers.

Cross-attention plays a crucial role in modeling interactions between image features and object queries, making it essential for accurate object detection. In the context of Latent Query Adversary (LQA), adversarial patches are introduced into the input image to interfere with the detection process. These patches generate adversarial tokens that are subsequently used as query inputs in the cross-attention computation. The resulting cross-attention output tokens are influenced by two primary components: (1) the image features obtained from the encoder, and (2) the object queries propagated through the residual connection. Notably, object queries are learnable parameters within the decoder and are not directly accessible or modifiable by an attacker.

**Figure 4:**
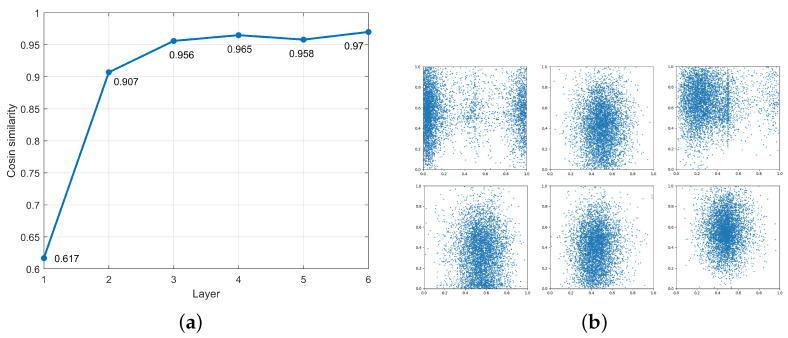
(**a**) Cosine similarity on different cross-attention layers. (**b**) Slot of object queries.

Prior studies have shown that residual components significantly influence the weight distribution of cross-attention outputs. To investigate this further, we conducted a comparative analysis of the cosine similarity between the output tokens and their corresponding residual components across different decoder layers in the DETR model. The results are visualized in Figure 4a.

Figure 4a illustrates that the cosine similarity between residual components and cross-attention outputs increases with the number of decoder layers. Adversarial patches primarily affect the cross-attention mechanism through encoder input tokens. A higher similarity indicates that the influence of adversarial tokens (from the encoder input) on the cross-attention output diminishes, implying a reduced adversarial effect.

The self-attention map in transformer-based detectors inherently contains both semantic foreground signals (e.g., object regions) and background clutter or texture noise (i.e., the residual component). During adversarial attack, perturbations that indiscriminately amplify all attention responses may waste energy on non-discriminative background regions, reducing transferability. To resolve this, we propose computing a joint attention matrix(JMA), which decomposes the cross-attention output into contributions from the encoder input tokens and the residual components. By suppressing the residual, we encourage the adversarial patch to interfere primarily with object-related feature pathways, thereby enhancing cross-model disruption while minimizing perceptual distortion.

Let $d\;=\;[d_{0},\;d_{1},\;\ldots ,\;d_{N}]$ denote the query inputs from the decoder in cross-attention, and let $e\;=\;[e_{0},\;e_{1},\;\ldots ,\;e_{M}]$ represent the key–value inputs from the encoder. The attention weight matrix for the *h*-th head in multi-head cross-attention can then be expressed as

(7) $$a^{h}\left(d_{m},e_{m}\right)=\frac{d_{n}W^{Q}{\left(e_{m}W^{K}\right)}^{T}}{\sqrt{d_{k}}}$$

The attention weight $a^{h}$ is multiplied with the value tokens and the corresponding projection matrix to produce the output token of the cross-attention mechanism, formulated as

(8) $$\sum_{h=1}^{H}W_{o}^{h}a^{h}W_{V}^{h}e\in R^{N\times M\times d_{k}}$$

where $W_{o}^{h}$ and $W_{v}^{h}$ denote the output and value projection matrices for the *h*-th attention head, respectively. The original input to the cross-attention module is retained as the residual component R, which is then concatenated with the computed cross-attention output. An $L_{1}$ normalization is applied along the concatenation dimension to construct the joint attention matrix (JAM), denoted as $J\in R^{N\times \left(M+1\right)\times d_{k}}$. The final JAM is defined as

(9) $$J={\mathrm{Norm}}_{L_{1}}\left(\mathrm{Concat}\left(\sum_{h=1}^{H}W_{o}^{h}A^{h}W_{v}^{h}e,\;R\right),\;\dim=1\right)$$

In J, the cross-attention output is explicitly decomposed into contributions from the encoder input and the residual component. However, directly increasing the weight of adversarial tokens within the JAM to amplify their influence in cross-attention computation is not practically feasible. This is due to the fact that positional encodings are embedded within each object query in the decoder, as illustrated in Figure 4b. By visualizing the spatial positions of detected objects associated with individual object queries, we observe a shared receptive region for objects detected by the same query.

Moreover, the position of the adversarial patch remains fixed in the input image. Although multiple adversarial tokens may be present in the encoder input, typically only one token is associated with each object query. Establishing a precise correspondence between adversarial tokens and object queries thus presents a non-trivial challenge.

To address this limitation, we propose an alternative strategy: suppressing the contribution of the residual component within the JAM. This approach effectively amplifies the relative influence of adversarial tokens on the cross-attention output, without requiring explicit alignment between object queries and adversarial regions. Based on this mechanism, we design a decoder-side adversarial loss, denoted as $L_{\mathrm{De}}$, to diminish the impact of the residual pathway and thereby enhance the adversarial effect. The detailed formulation of $L_{\mathrm{De}}$ is presented in Equation (10).

(10) $$L_{\mathrm{De}}=log\left(\sum J\left[:,-1\right]\right)$$

#### 3.2.3. Total Loss

In addition to $L_{\mathrm{En}}$ and $L_{\mathrm{De}}$, the LQA incorporates two additional loss components: a classification loss $L_{\mathrm{cls}}$ and a total variation loss $L_{\mathrm{TV}}$, defined as follows:

(11) $$L_{\mathrm{cls}}=s_{\mathrm{adv}}-1$$

Here, $s_{\mathrm{adv}}$ denotes the confidence score assigned to the target class by the model. In our formulation, the background class is selected as the target class to encourage misclassification toward non-object predictions.

(12) $$L_{TV}=\sum_{i,j}\sqrt{{\left(x_{i,j-1}-x_{i.j}\right)}^{2}+{\left(x_{i+1,j}-x_{i,j}\right)}^{2}}$$

The total variation loss $L_{\mathrm{TV}}$ penalizes spatial discontinuities between adjacent pixels in the adversarial patch, promoting smoothness. A smoother patch is more robust to real-world noise and better mimics natural textures.

In total, LQA integrates four distinct loss components. Determining appropriate weighting coefficients for these losses presents a significant challenge, especially due to potential conflicts during optimization—for instance, between $L_{\mathrm{cls}}$ and $L_{\mathrm{TV}}$. To address this issue, we adopt an adaptive weighting strategy inspired by Liu et al. [38], which dynamically adjusts the loss coefficients based on their historical variations across iterations.

Specifically, the relative change in each loss $L_{k}$ between consecutive iterations is computed as

(13) $$w_{k}\left(t-1\right)=\frac{L_{k}\left(t-1\right)}{L_{k}\left(t-2\right)}$$

Then, the normalized weight $\lambda_{k}\left(t\right)$ for the *k*-th loss at iteration *t* is calculated using a softmax-like function:

(14) $$\lambda_{k}\left(t\right)=\alpha \cdot \frac{e^{w_{k}\left(t-1\right)}}{e^{\sum_{K}w_{k}\left(t-1\right)}}$$

where α controls the overall magnitude of the weighted losses and is empirically set to 10 in our experiments. Finally, the overall objective function used in LQA is formulated as

(15) $$L_{Overall}=\sum_{k=0}^{K}\lambda_{k}\cdot L_{k}+L_{TV},L_{k}\in \left[L_{En},L_{De},L_{cls}\right]$$

### 3.3. Experimental Settings

Detector. This paper employs DETR as the surrogate white-box detector for generating adversarial patches. In addition, we use Anchor-DETR, DAB-DETR, Conditional-DETR, Deformable-DETR, and DINO as black-box detectors to evaluate the transferability of the generated adversarial examples.

Although all these models are based on the transformer architecture, they differ significantly in structure and design. Conducting experiments in a black-box setting with these diverse architectures enables a comprehensive assessment of the generalization and effectiveness of adversarial attacks across different variants of transformer-based object detectors.

Datasets and Evaluation Metrics. We conduct experiments on two widely used datasets: the INRIA Person dataset and the COCO Person dataset.

The INRIA Person dataset is a benchmark for pedestrian detection, containing thousands of images, including 614 positive training samples and 288 positive test samples. The COCO dataset is a large-scale object detection benchmark consisting of 80 object categories, with 118 K training images and 5 K validation images. The COCO Person dataset, a subset of the COCO validation set, contains 1695 images that include at least one person.

Both datasets encompass a wide variety of scenes, including indoor and outdoor environments, and varying lighting conditions, times of day, and weather scenarios. Furthermore, the datasets feature a broad range of human poses and appearances, making them well-suited for evaluating the performance of adversarial patches under diverse real-world conditions.

In our experiments, adversarial patches are trained on the INRIA Person training set and evaluated on both the INRIA Person and COCO Person validation sets. Detailed experimental settings and results are summarized in Table 1.

In object detection, mAP (mean average precision) is a widely adopted evaluation metric that quantifies the precision of a detector across different object categories. In our experiments, the effectiveness of adversarial attacks is evaluated by measuring the reduction in mAP@0.5—that is, the average precision at an Intersection over Union (IoU) threshold of 0.5—when adversarial examples are introduced.

The evaluation of detection performance typically involves two key criteria: localization accuracy and classification consistency. Localization accuracy is determined by whether the predicted bounding box overlaps sufficiently with the corresponding ground truth box, as measured by the IoU metric and compared against a predefined threshold. Classification consistency evaluates whether the predicted class label matches the true object category. A detection is considered correct only if both criteria are satisfied.

Based on these criteria, positive detections are identified, and precision is computed accordingly. The average precision (AP) is then calculated for each object category by integrating the precision–recall curve at different confidence thresholds. In this work, we focus specifically on the person category; hence, the reported mAP corresponds to the AP of the person class under the IoU threshold of 0.5.

**Figure 5:**
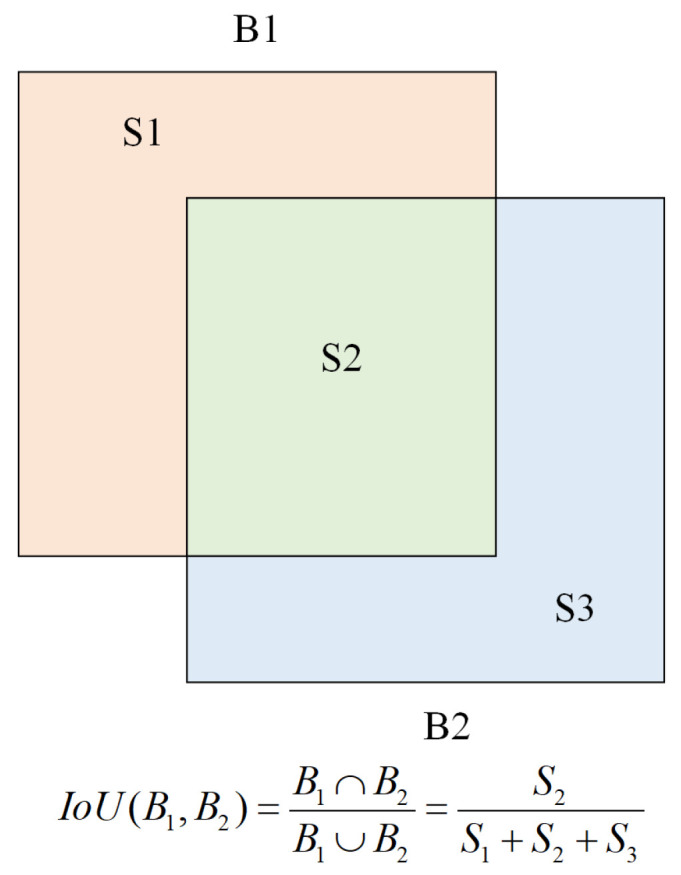
Illustration of the Intersection over Union (IoU) calculation. Given two bounding boxes $B_{1}$ and $B_{2}$, IoU is defined as the ratio of their intersection area to their union area. An IoU of 1 indicates perfect overlap, although such a scenario is rarely achieved in practice. A commonly used threshold for considering a detection as correct is IoU ≥ 0.5.

As illustrated in Figure 5, the IoU between two bounding boxes $B_{1}$ and $B_{2}$ is defined as the ratio of their intersection area to their union area:

$$\mathrm{IoU}\left(B_{1},B_{2}\right)=\frac{\mathrm{Area}(B_{1}\cap B_{2})}{\mathrm{Area}(B_{1}\cup B_{2})}$$

While an IoU of 1 ideally indicates perfect alignment between the predicted and ground truth boxes, achieving this is rare in practice. Therefore, a detection is typically considered valid if its IoU with the ground truth exceeds a certain threshold—commonly set to 0.5.

Implementation Details and Comparative Methods. All experiments are implemented using PyTorch 1.10 on an NVIDIA GeForce RTX 3090 GPU. For a fair comparison, all adversarial patch generation methods are implemented with DETR as the surrogate detector.

We employ the Adam optimizer with an initial learning rate of 0.03. The learning rate is decayed by a factor of 0.97 when the change in total loss falls below $1\times 10^{-4}$ over consecutive iterations. Input images are resized such that the longest side is 416 pixels, and then zero-padded to obtain a fixed size of 416 × 416 pixels. The batch size is set to 32, and the model is trained for a total of 500 epochs.

The adversarial patch is initialized with a size of 300 × 300 pixels. During testing, it is scaled to 0.13 times the height of the detected bounding box and centered on the target object. This scaling strategy results in patches of varying sizes depending on the scale of the target object, enabling more realistic evaluation across different object dimensions.

The comparative methods include Adversarial Patch (AdvPatch) [13], Transfer-based Self-Ensemble Attack (T-SEA) [32], Patch-Fool [19], Gradient Normalization Scaling (GNS) [37], and Pay No Attention (PNA) [34]. A detailed overview of these methodologies is provided in Table 2.

Among these, AdvPatch serves as the baseline method in our study. T-SEA enhances attack transferability through a self-ensemble strategy that aggregates predictions across multiple augmented views of the input. Patch-Fool is specifically designed for transformer-based models and aims to disrupt attention scores between the adversarial patch and other image regions. GNS improves the stability of the optimization process by scaling the backpropagated gradients, thereby enhancing the generalization of adversarial examples across different models. PNA further improves transferability by suppressing gradient propagation through the attention modules during backpropagation.

All methods have been adapted to train with DETR as the surrogate model. Among them, Patch-Fool, GNS, and PNA are explicitly tailored for attacking transformer-based architectures.

### 3.4. Experimental Results

The experimental results are summarized in Table 3 and Table 4, which report the performance of various adversarial patch generation methods under black-box settings across two datasets: INRIA Person and COCO Person.

We compare the average precision decline (i.e., mAP@0.5 drop) achieved by different attack methods. AdvPatch perturbs the detector by increasing background scores across all detection outputs, resulting in a precision reduction of 30.11% on the INRIA Person dataset and 23.48% on the COCO Person dataset.

T-SEA enhances transferability through extensive data augmentation and gradient manipulation during backpropagation. These augmentations include variations in brightness, contrast, saturation, and hue, as well as random rotation and occlusion of the adversarial patch. Additionally, T-SEA employs the ShakeDrop technique to fine-tune the backbone network during training, promoting better gradient aggregation and self-ensembling. Compared with AdvPatch, T-SEA achieves a 7.16% improvement on the INRIA Person dataset and a 3.27% improvement on the COCO Person dataset. However, its performance remains inferior to that of transformer-specific approaches such as PNA and GNS.

PNA and GNS are both tailored for attacking transformer-based models and primarily focus on manipulating gradient signals. GNS observes that mild gradients can significantly impact transferability and thus applies channel-wise normalization to scale them accordingly. In contrast, PNA suppresses gradient propagation through attention matrices during training, effectively reducing model robustness to adversarial perturbations. Both methods achieve notable improvements in cross-model generalization. As shown in Table 3, their performances are comparable; however, neither matches the effectiveness of LQA. It is worth noting that LQA differs fundamentally from PNA. PNA suppresses gradients in attention maps during inference to enhance model robustness. In contrast, LQA constructs a differentiable proxy $\hat{A}$ of self-attention to guide perturbation toward maximizing detection failure. Thus, while both involve attention manipulation, their implementation paradigms (optimization guidance vs. attention modification) are orthogonal.

Patch-Fool, like LQA, targets the self-attention mechanisms within the encoder. However, instead of focusing on local regions, Patch-Fool disrupts global attention scores, leading to broader influence over predictions. Compared to AdvPatch, it improves transferability by 4.65% on the INRIA Person dataset and 5.37% on the COCO Person dataset. To quantitatively validate the advantage of LQA’s localization, we compare it with Patch-Fool using the Average Response Ratio (ARR), defined as the ratio of average attention gain on foreground tokens to that on background tokens.

(16) $$\mathrm{ARR}=\frac{\mathrm{Avg}.\;\mathrm{attention}\;\mathrm{gain}\;\mathrm{on}\;\mathrm{foreground}\;\mathrm{tokens}}{\mathrm{Avg}.\;\mathrm{attention}\;\mathrm{gain}\;\mathrm{on}\;\mathrm{background}\;\mathrm{tokens}}$$

LQA achieves an ARR of 4.8, significantly higher than Patch-Fool’s 1.3, indicating that its perturbation is more focused on relevant object regions, demonstrating that our localized strategy effectively suppresses spurious responses in background areas. This confirms that localization mitigates the collateral interference inherent in global methods, thereby enhancing both precision and transferability of the attack.

LQA shifts the focus from global attention disruption to local self-attention score manipulation, thereby enhancing its ability to perturb foreground features via adversarial patches. Furthermore, we strengthen the effect of adversarial patches on cross-attention mechanisms by leveraging joint attention maps. Consequently, compared to the AdvPatch baseline, LQA achieves improvements of 27.56% and 10.34% on the two datasets, respectively. Compared to the second-best-performing method, GNS, LQA demonstrates gains of 18.38% and 4.97%, respectively.

We optimize LQA with COCO dataset-pretrained detectors and evaluate its performance on both the INRIA Person and COCO Person test sets. Given the smaller size of the INRIA dataset compared to COCO, results on the COCO dataset serve to illustrate how different methods perform under training and test data distribution discrepancies. Our findings demonstrate that despite domain shifts, LQA achieves a 4.97% performance improvement over the second-ranked method, affirming its superior attack transferability.

### 3.5. Ablation Experiments

To investigate the contribution of different loss components to the transferability of LQA, we conduct ablation experiments on the INRIA Person dataset. AdvPatch is used as the baseline method. We progressively enhance AdvPatch by incorporating two key components—the encoder-focused loss $L_{\mathrm{En}}$ and the decoder-focused loss $L_{\mathrm{De}}$—leading to the full LQA framework. The results of these ablation studies are summarized in Table 5.

AdvPatch generates adversarial patches primarily through data augmentation and by increasing background confidence scores in the detector outputs. In comparison, $L_{\mathrm{De}}$ introduces perturbations to the cross-attention mechanism within the decoder using joint attention matrices, leading to a moderate improvement in attack transferability over AdvPatch. The $L_{\mathrm{En}}$ loss further enhances transferability by introducing localized disruptions to the self-attention mechanism, resulting in an improvement of over 20% compared to the AdvPatch baseline.

When both components are integrated into the proposed LQA framework, the combined loss formulation yields a substantial performance gain over using either loss individually, demonstrating the effectiveness of our unified optimization strategy.

To validate the necessity of adaptive weighting, we compare it against single loss (AdvPatch) and equal weights in Table 6. Our method reduces mAP by 19.36 compared to equal weighting, demonstrating that dynamic balancing of $L_{\mathrm{En}}$ and $L_{\mathrm{De}}$ is crucial for optimal attack performance.

### 3.6. Detection Results

This subsection presents the detection results of LQA across various transformer-based object detectors.

**Figure 6:**
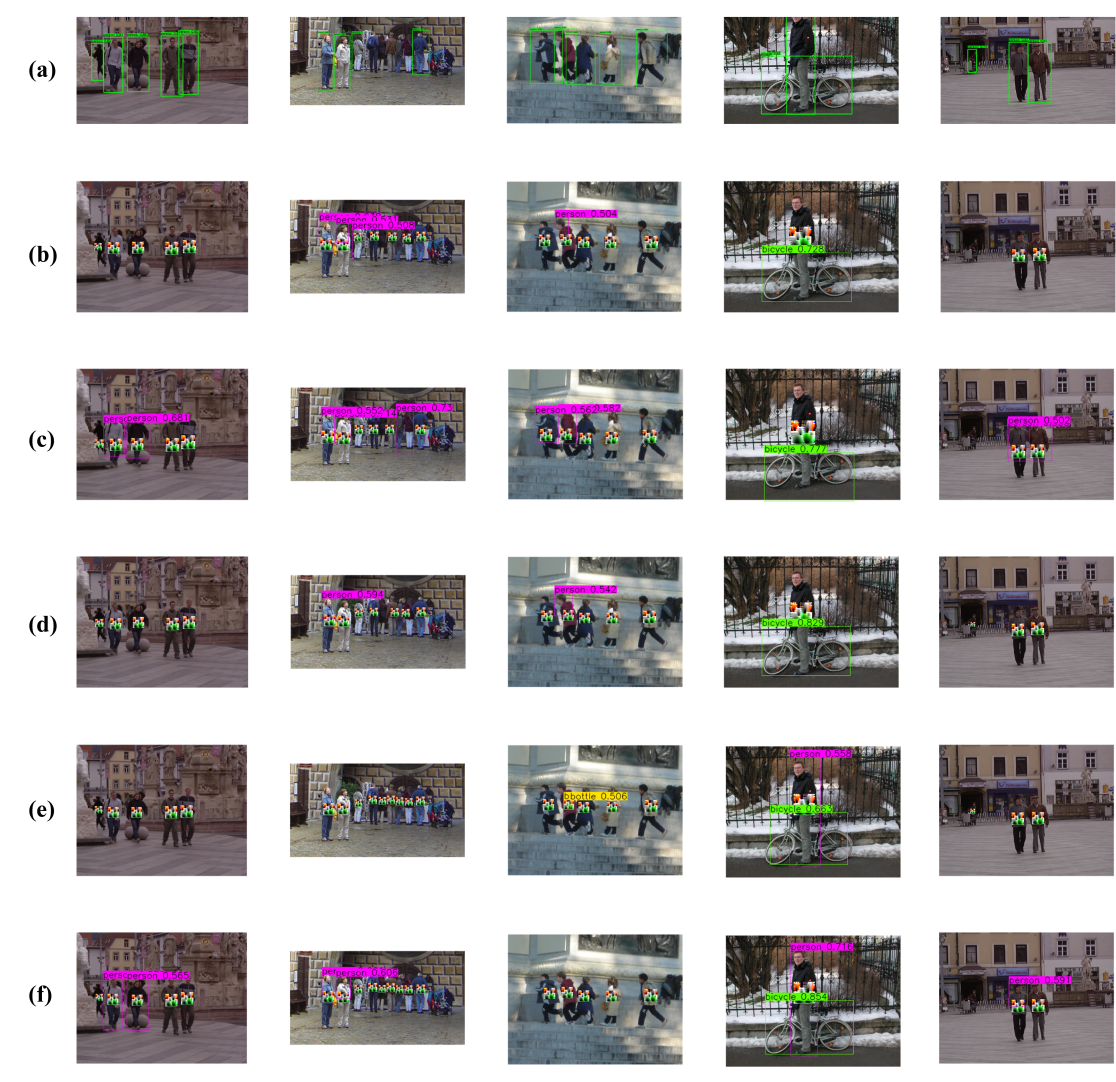
Detection results. (**a**) Original results. (**b**) Anchor-DETR. (**c**) DAB DETR. (**d**) Conditional-DETR. (**e**) Deformable-DETR. (**f**) DINO.

Figure 6a displays the original detection results without any adversarial perturbation, while Figure 6b–f illustrate the detection outputs when the adversarial patch generated by LQA is applied to Anchor-DETR, Conditional-DETR, DAB-DETR, Deformable-DETR, and DINO, respectively. The figure clearly demonstrates that LQA induces missed detections across all evaluated models, with varying degrees of impact depending on the detector architecture.

### 3.7. Real-World Verification

In this section, we conduct real-world validation experiments using Anchor-DETR and Deformable-DETR to evaluate the practical effectiveness of LQA in sensor-driven AI environments. Video footage is captured using a Xiaomi 14 Pro smartphone—a representative consumer-grade imaging sensor widely deployed in IoT and mobile perception systems—to emulate realistic input conditions faced by vision sensors in the wild. Prior to attack evaluation, we verify the baseline detection accuracy of the models under normal operating conditions. This setup allows us to assess how adversarial patches impact detector reliability when processed through real optical and digital sensor pipelines, where factors such as dynamic range, auto-exposure, and lens distortion inherently shape the input data.

We then apply the LQA-generated adversarial patch to a tablet screen placed within the scene, continuously recording the detector outputs during the interaction. Detections are considered valid if their confidence score exceeds a threshold of 0.7, consistent with the default settings of the detectors.

**Figure 7:**
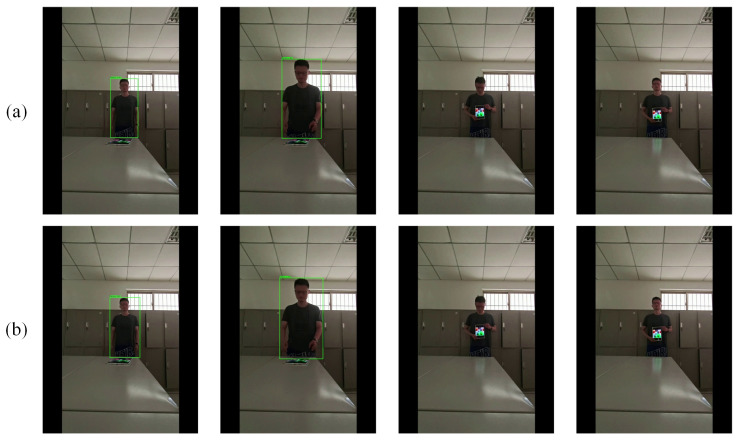
Real-world verification. (**a**) Anchor-DETR. (**b**) Deformable-DETR. The detection results at frames at 1, 4, 9, and 12 s, arranged from left to right, are displayed.

The real-world detection results are presented in Figure 7. https://www.bilibili.com/video/BV1vUhVeiE18/?spm_id_from=333.999.0.0, accessed on 4 January 2026.

The transferability results across five heterogeneous transformer-based visible-object detectors, together with the comparison of real-world performance, demonstrate the practical viability of LQA.

## 4. Summary and Perspective

This paper introduces LQA, an adversarial patch attack method specifically designed for transformer-based object detectors—architectures now commonly deployed in resource-constrained vision sensors for autonomous navigation, smart surveillance, and human–machine interaction. By simultaneously disrupting both encoder and decoder attention mechanisms, LQA not only achieves state-of-the-art transferability but also reveals fundamental security gaps in attention-based feature aggregation that underpin modern edge-AI sensing platforms.

Extensive experiments conducted on two benchmark datasets and across five state-of-the-art transformer-based detection models demonstrate the superior transferability of LQA. In addition, real-world experiments are carried out to validate its effectiveness in physical environments.

During these real-world evaluations, we observe that environmental factors such as lighting conditions and viewing angles significantly affect the performance of LQA. Moreover, the choice of physical medium used to display the adversarial patch also plays a critical role in determining its efficacy. Future work will focus on further enhancing the robustness of LQA under practical conditions and adapting LQA to emerging variants of transformer-based architectures.

## Figures and Tables

**Table 1:** The black-box detectors in experiment.

Detectors	Backbones	Main Differences from DeTR	mAP@0.5 in INRIA Person	mAP@0.5 in COCO Person
Anchor-DETR	ResNet50	Row-Column Decoupled Attention	80.87	63.62
DAB-DETR	ResNet50	Learnable anchor boxes in Decoder	79.81	64.83
Conditional-DETR	ResNet50	Conditional cross attention	75.04	51.07
Deformable-DETR	ResNet50	Multi-scale deformable attention	81.19	65.64
DINO	Swim Transformer	Contrastive DeNoising Training and Mixed Query Selection	84.98	64.89

**Table 2:** The comparison methods in the experiment.

Method	Data Augmentation	Advanced Gradient	Designed for Transformer-Based Models
AdvPatch	**✓**		
T-SEA	**✓**	**✓**	
Patch-Fool	**✓**		**✓**
GNS	**✓**	**✓**	**✓**
PNA	**✓**	**✓**	**✓**
LQA	**✓**		**✓**

**Table 3:** Results on INRIA Person dataset.

Attack Method	Anchor	DAB	Conditional	Deformable	DINO	Avg
AdvPatch	32.74	26.35	38.29	36.48	16.68	30.11
T-SEA	36.70	34.60	55.52	41.25	18.28	37.27
Patch-Fool	33.75	33.88	56.30	42.54	12.53	35.76
PNA	31.11	29.41	57.38	49.08	23.50	38.10
GNS	34.41	37.47	57.26	45.19	22.13	39.29
LQA	64.38	60.37	68.53	56.62	38.45	57.67

**Table 4:** Results on COCO Person dataset.

Attack Method	Anchor	DAB	Conditional	Deformable	DINO	Avg
AdvPatch	24.90	25.06	21.14	25.10	21.22	23.48
T-SEA	29.10	29.01	30.59	26.20	18.87	26.75
Patch-Fool	31.27	28.45	28.64	26.00	20.76	27.02
PNA	31.26	27.85	32.02	29.30	22.89	28.66
GNS	31.65	30.78	31.02	28.17	22.63	28.85
LQA	47.95	43.35	36.08	37.93	30.81	33.82

**Table 5:** The contribution of different losses in LQA.

Loss	Anchor	DAB	Conditional	Deformable	DINO	Avg
AdvPatch	32.74	26.35	38.29	36.48	16.68	30.11
w/$L_{De}$	35.28	30.15	44.67	40.50	21.47	34.41
w/$L_{En}$	56.44	61.20	55.52	49.28	33.55	51.98
LQA	64.38	60.37	68.53	56.62	38.45	57.67

**Table 6:** Ablation study on loss weighting strategies.

Strategy	$L_{\mathrm{En}}$	$L_{\mathrm{De}}$	$L_{\mathrm{cls}}$	Avg
Single loss	0	0	1.0	30.11
Equal weights	1.0	1.0	1.0	38.41
Ours (adaptive)	-	-	-	57.67

## Data Availability

The raw data supporting the conclusions of this article will be made available by the authors on request.
